# Effects of heat stress on carbohydrate and lipid metabolism in growing pigs

**DOI:** 10.14814/phy2.12315

**Published:** 2015-02-25

**Authors:** M Victoria Sanz Fernandez, Jay S Johnson, Mohannad Abuajamieh, Sara K Stoakes, Jacob T Seibert, Lindsay Cox, Stanislaw Kahl, Theodore H Elsasser, Jason W Ross, S Clay Isom, Robert P Rhoads, Lance H Baumgard

**Affiliations:** 1 Department of Animal Science, Iowa State UniversityAmes, Iowa, USA; 2 Animal Biosciences and Biotechnology Laboratory, Agricultural Research Service, US Department of AgricultureBeltsville, Maryland, USA; 3 Department of Animal, Dairy and Veterinary Sciences, Utah State UniversityLogan, Utah, USA; 4 Department of Animal and Poultry Sciences, Virginia TechBlacksburg, Virginia, USA

**Keywords:** Epinephrine challenge, glucose tolerance test, heat stress, metabolism, thyroid hormones

## Abstract

Heat stress (HS) jeopardizes human and animal health and reduces animal agriculture productivity; however, its pathophysiology is not well understood. Study objectives were to evaluate the direct effects of HS on carbohydrate and lipid metabolism. Female pigs (57 ± 5 kg body weight) were subjected to two experimental periods. During period 1, all pigs remained in thermoneutral conditions (TN; 20°C) and were *ad libitum* fed. During period 2, pigs were exposed to: (1) constant HS conditions (32°C) and fed *ad libitum* (*n* = 7), or (2) TN conditions and pair-fed (PFTN; *n* = 10) to minimize the confounding effects of dissimilar feed intake. All pigs received an intravenous glucose tolerance test (GTT) and an epinephrine challenge (EC) in period 1, and during the early and late phases of period 2. After 8 days of environmental exposure, all pigs were killed and tissue samples were collected. Despite a similar reduction in feed intake (39%), HS pigs tended to have decreased circulating nonesterified fatty acids (NEFA; 20%) and a blunted NEFA response (71%) to the EC compared to PFTN pigs. During early exposure, HS increased basal circulating C-peptide (55%) and decreased the insulinogenic index (45%) in response to the GTT. Heat-stressed pigs had a reduced T_3_ to T_4_ ratio (56%) and hepatic 5′-deiodinase activity (58%). After 8 days, HS decreased or tended to decrease the expression of genes involved in oxidative phosphorylation in liver and skeletal muscle, and ATGL in adipose tissue. In summary, HS markedly alters both lipid and carbohydrate metabolism independently of nutrient intake.

## Introduction

Heat stress (HS) is a major environmental hazard for both humans and animals. Heat claims more human lives than all other climatic events combined (Changnon et al. 1996), with the young and elderly populations being the most susceptible (Leon and Helwig 2010). Surprisingly, despite increased understanding on the pathophysiology of heat-related illnesses (Bouchama and Knochel 2002), the only standard procedures to treat heat victims are cooling and rehydration (Leon and Helwig 2010).

In addition to morbidity and mortality, HS negatively impacts livestock productivity. Environmental hyperthermia costs global animal agriculture several billion dollars annually due to reduced and inconsistent growth, decreased carcass quality, compromised reproduction, reduced milk yield and egg production, and increased veterinary costs (St-Pierre et al. 2003; Baumgard and Rhoads 2013). We have previously reported that, despite hypercatabolic hallmarks like marked hypophagia and weight loss, HS increases basal and stimulated (i.e., in response to a metabolic challenge) circulating insulin and decreases adipose tissue mobilization in a variety of species (Baumgard and Rhoads 2013), including pigs (Pearce et al. 2013a). Interestingly, diabetic humans and rodents are more susceptible to heat-related illnesses and exogenous insulin rescues this phenotype (Semenza et al. 1999; Niu et al. 2003). Furthermore, thermal therapy improves insulin sensitivity in diabetic and obese rodents (Kokura et al. 2007; Gupte et al. 2009) and humans (Hooper 1999). Collectively, these reports suggest that insulin or altered insulin action and subsequent shifts in metabolism are a conserved response among species that might play an important role in the adaptation and survivability to HS (Baumgard and Rhoads 2013).

Thus, the first study objective was to determine the effects of HS on the temporal responses to acute metabolic challenges in pigs. Our second objective was to investigate the systemic and molecular changes responsible for the HS-induced shift in postabsorptive metabolism. We hypothesized that HS has a direct effect on metabolism, independent of the heat-induced hypophagia. The pig is both a relevant biomedical model (Prather et al. 2013) and an important agricultural species, thus results will likely have important implications in both human health and animal production.

## Materials and Methods

### Animals and experimental design

Iowa State University Institutional Animal Care and Use Committee approved all procedures involving animals. Seventeen crossbred female pigs (57 ± 5 kg body weight) were randomly assigned to 1 of 2 treatments during two experimental periods. Prepubertal female pigs were utilized to be consistent with our previous experiments (Sanz-Fernandez et al. 2012; Pearce et al. 2013a,b; Sanz Fernandez et al. 2014). During period 1 (4 days in length), all pigs were exposed to thermoneutral conditions [TN; 20°C, ∼35% humidity (Federation of Animal Sciences Societies 2010)] and fed *ad libitum*. During period 2 (8 days in length), pigs were either exposed to constant HS conditions (32°C, ∼23% humidity (Federation of Animal Sciences Societies 2010); *n* = 7) and fed *ad libitum* or remained in TN conditions and were pair-fed (PFTN; *n* = 10) to their HS counterparts, in order to eliminate the confounding effect of dissimilar nutrient intake. For the pair-feeding calculations, as-fed period 1 daily feed intake (FI) was averaged for each pig and used as a baseline. For each HS pig, the decrease in intake during period 2 was calculated as the percentage of FI reduction relative to period 1 for each day of HS exposure. This percentage of FI reduction was averaged for all the HS pigs per day of exposure and applied individually to the baseline of each PFTN pig. The calculated amount of feed was offered to the PFTN pigs three times a day (∼0800, 1400, and 2100 h) in an attempt to minimize large postprandial shifts in metabolism. All pigs were fed a standard swine grower diet consisting mainly of corn and soybean meal formulated to meet or exceed nutrient requirements (National Research Council 2012). Pigs were individually housed in metabolic crates in 1 of 6 environmental chambers where temperature was controlled but humidity was not governed. Both parameters were recorded every 30 min by a data logger (Lascar EL-USB-2-LCD, Erie, PA). Rectal temperature was measured with a digital thermometer (ReliOn, Waukega, IL), respiration rate was determined by counting flank movements, and both indices were measured twice a day (0700 and 2200 h) and condensed into daily averages. Pigs were killed on d 8 of period 2 using the captive bolt technique and subcutaneous adipose tissue (AT) from the cranial dorsum, skeletal muscle from longissimus dorsi (LD), liver, and pancreas samples were immediately collected and snap frozen or preserved for histology.

### Blood sampling and metabolic challenges

An indwelling jugular catheter was surgically inserted on d 1 of period 1 in all pigs. Pigs were anesthetized with a mixture of tiletamine/zolazepam (Telazol®; Fort Dodge Laboratories Inc., Fort Dodge, IA), ketamine (Ketaject®; Clipper Distributing Company, LLC, St Joseph, MO), and xylazine (Anased®; Lloyd Inc., Shenandoah, IA) for a final concentration of 100, 50, and 50 mg/mL, respectively. Pigs were injected IM with the anesthetics mixture at 1 mL/23 kg BW dose. With the pigs in dorsal recumbency, the jugular vein was located with a 14 G, 3.75 cm introducer needle (Mila International Inc., Erlanger, KY) using a percutaneous technique. Once in the vein, Tygon® tubing (1.016 mm inside diameter, 1.778 mm outside diameter; Saint-Gobain Performance Plastic Corp., Aurora, OH) was inserted through the introducer needle (15 cm). After removing the introducer, a small skin incision was made cranial to the tube's insertion point exposing the subcutaneous tissue. Using laparoscopic forceps (Auto Suture Endo Grasp™ 5 mm; Covidien, Mansfield, MA), the tube was tunneled subcutaneously from the incision and exteriorized at the dorsum of the back. The neck incision was sutured and the catheter remained subcutaneous and not exposed. All pigs received antibiotics (Ceftiofur, Excede®; Pfizer Animal Health, New York, NY) and nonsteroidal antiinflammatory drugs (Flunixin meglumine, Banamine-S; Schering-Plough Animal Health Corp., Whitehouse Station, NJ) during the surgery. Intravenous antibiotic therapy continued daily for the rest of the study (Ampicillin, Polyflex®; Fort Dodge Laboratories Inc., Fort Dodge, IA) and catheters were flushed twice daily with heparinized saline (100 U/mL).

Animals were fasted for 2 h prior to the metabolic challenges and daily blood sampling (basal metabolism samples). Glucose tolerance tests (GTT) were performed on d 3 of period 1 and d 1 and 6 of period 2. A 50% dextrose solution bolus (VetOne®; MWI Veterinary Supply, Boise, ID) was administered at 0.5 g/kg BW dose via the jugular catheter. Blood samples were collected at −20, −10, 0, 5, 7.5, 10, 15, 20, 30, 45, 60 min relative to the glucose administration. Epinephrine challenges (EC; 3 *μ*g/kg BW; American Regent, Inc., Shirley, NY) were performed on d 4 of period 1 and d 2 and 7 of period 2 and blood samples were collected at −20, −10, 0, 2.5, 5, 7.5, 10, 15, 20, 30, 45, 60 min relative to the epinephrine administration. Daily blood samples were obtained at 0800 h so animals in both treatments were in a preprandial state. All blood samples were collected into disposable glass tubes containing 250 U of sodium heparin and immediately placed on ice. Plasma was harvested by centrifugation at 1300 × *g* for 15 min at 4°C and stored at −80°C for later analysis.

### Blood parameters analyses

Plasma glucose and nonesterified fatty acids (NEFA) concentrations were measured enzymatically using commercially available kits (Wako Chemicals USA, Richmond, VA). The intra and interassay coefficients of variation were 4.3 and 3.6%, and 7.3 and 5.8% for glucose and NEFA, respectively. Plasma *β*-hydroxybutyrate (BHB) concentration was also analyzed enzymatically using a commercially available kit (Pointe Scientific Inc., Canton, MI) and the intra and interassay coefficients of variation were 3.2 and 2.2%. Plasma insulin and C-peptide concentrations were analyzed using ELISA kits (Mercodia AB, Uppsala, Sweden) following the manufacturer's instructions. The intra and interassay coefficients of variation were 2.9 and 6.3%, and 7.2 and 4.9% for insulin and C-peptide, respectively. Plasma lipopolysaccharide (LPS)-binding protein (LBP) concentration was determined using an ELISA kit (Hycult® biotech, Uden, The Netherlands) and the intraassay coefficients of variation was 1.5%. Total plasma thyroxine (T_4_) and triiodothyronine (T_3_) concentrations were evaluated using commercially available solid phase RIA kits (MP Biomedicals, LLC; Irvine, CA) according to the manufacturer's instructions. The assay kits were validated (recovery and linearity of diluted samples) for use with porcine plasma samples as previously described (Kahl et al. 2000), and the intraassay coefficients of variation were 3.7 and 4.1% for T_3_ and T_4_, respectively.

### Hepatic 5′-deiodinase type I (5′D) activity

Outer-ring deiodinating activity was determined by quantifying the ^125^I^-^ source released from 3,3′,5′-[^125^I]-T_3_ (rT_3_) as previously described (Kahl et al. 2000). In brief, hepatic samples were homogenized in 0.01 mol/L HEPES buffer (pH 7.0, 0.25 mol/L sucrose, 5 mmol/L EDTA). After centrifugation (30 min at 2000 × *g*), the supernatant was incubated for 5 min in 0.1 mol/L phosphate buffer (pH 7.0, 1 mmol/L EDTA) in the presence of 5 mmol/L dithiothreitol at 37°C with approximately 80,000 cpm of [^125^I]-rT_3_ (DuPont-New England Nuclear, Boston, MA) and 500 nmol/L of unlabeled rT_3_ (Calbiochem, La Jolla, CA). The released ^125^I^−^ source was isolated as trichloroacetic acid-soluble radioactivity. The 5′D activity was expressed as nmol of I^−^ produced per mg protein per hour. Protein concentration in homogenates was determined by bicinchoninic acid assay (BCA, Pierce™; Thermo Fisher Scientific Inc., Rockford, IL). The intraassay coefficient of variation was 8.6%.

### Pancreatic insulin content

Pancreatic protein was extracted at 4°C overnight with acid-ethanol (75% ethanol, 1.5% HCl). After centrifugation at 2000 rpm for 15 min, the supernatant was neutralized with 1 mol/L Tris (pH = 7.5) and total protein was determined by BCA assay (Pierce™; Thermo Fisher Scientific Inc., Rockford, IL). Neutralized samples were further diluted (1:20000) and insulin concentration was measured using an ELISA kit (Mercodia AB, Uppsala, Sweden) following the manufacturer's instructions. Pancreatic insulin content was calculated relative to the total amount of protein.

### Pancreatic immunohistochemistry

Pancreatic tissue samples were fixed in 10% formalin for 24 h and then transferred into 70% ethanol. Fixed samples were sectioned at 4 *μ*m thickness. Two random sections per pig were stained for insulin using an indirect immunoperoxidase technique. The primary and secondary antibodies (Dako, Carpinteria, CA) were a polyclonal guinea pig antiporcine insulin antibody (Dilution 1:1000) and a polyclonal rabbit antiguinea pig horse radish peroxidase-conjugated immunoglobulin (Dilution 1:50), respectively. Diaminobenzidine (Dako, Carpinteria, CA) was used as the chromogen and Mayer's hematoxylin as the counterstain. Negative controls were generated by omitting the primary or the secondary antibody. Five nonoverlapping fields per section were imaged at 50× using Q-capture Pro 6.0 software (Qimaging®, Surey, BC) with the operator being blind to the treatments. The relative-stained area was measured by thresholding and the number and size of insulin-stained cell clusters were determined using the particle analysis tool in ImageJ (US National Institutes of Health, Bethesda, MD). The results obtained in each section were averaged into a single measurement per pig.

### RNA isolation and quantitative PCR

Total RNA was extracted from AT, LD and liver using TRIzol® Reagent (Invitrogen, Carlsbad, CA) following the manufacturer's protocol and was utilized for cDNA synthesis using the QuantiTect Reverse Transcription Kit (Qiagen, Hilden, Germany). Gene expression differences were determined using qPCR (BioMark™ System, Fluidigm Corporation, San Francisco, CA) on 40, 44, and 44 genes for AT, LD, and liver, respectively (Tables5). Genes chosen for analysis were selected based on the RNA-Seq output of a similar experiment (Seibert et al. 2014). Expression normalization across samples within a tissue was performed by calculating a delta Ct (Δ*C*_t_) value for each sample using *GAPDH* for AT, *β-actin* for LD, and *RPL32* in liver, as transcript abundance for these genes proved to be the most similar between treatments for each given tissue (*P *>* *0.05). ΔΔ*C*_t_ values were calculated utilizing a reference sample and fold differences between treatments were obtained by applying the equation 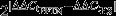, where a positive and a negative value indicate an increase and a decrease in transcript abundance, respectively, in HS pigs relative to PFTN controls. Statistical analysis was performed on the ΔΔ*C*_t_ values and data are reported in the results section as relative fold difference.

### Calculations and statistical analysis

Metabolic responses to the GTT and EC were calculated as area under the curve (AUC) by linear trapezoidal summation between successive pairs of metabolite concentrations and time coordinates after subtracting baseline values. Glucose and insulin AUC in response to the GTT were calculated through min 20 of the challenge. Glucose and NEFA responses to the EC were determine through min 30 and 15, respectively. For the GTT, glucose and insulin deltas were calculated for each challenge as the change in their concentration between 0 and 5 min, and 0 and 7.5 min, respectively. In addition, glucose disappearance was calculated as the slope of glucose concentrations between 5 and 30 min. An insulinogenic index was determined as the insulin AUC to glucose AUC ratio.

All data were statistically analyzed using SAS version 9.3 (SAS Institute Inc., Cary, NC). Single measurements were analyzed using PROC MIXED and the model included treatment as fixed effect. Variables with multiple measurements per pig over time were analyzed using PROC MIXED with day of period 2 as the repeated effect and period 1 values used as a covariate. Auto regressive and spatial power covariance structures were utilized for equally and unequally spaced measurements, respectively. The model included treatment, day and their interaction as fixed effects. For each variable, normal distribution of residuals was tested using PROC UNIVARIATE and logarithmic transformation was performed when necessary. Data are reported as least square means and considered significant if *P *≤* *0.05 and a tendency if 0.05 < *P *≤* *0.10.

## Results

As expected, during period 2 HS pigs had increased rectal temperature and respiration rate (1.5°C and 4.5 fold, respectively; *P *<* *0.01) compared to PFTN controls (Table1). Heat stress decreased FI (39%, *P *<* *0.01), and by design, PFTN pigs’ FI was reduced similarly (Table1). Overall, this data indicate that pigs experienced an acute and sustained heat load indicative of marked heat stress.

**Table 1:** Effects of pair-feeding or heat stress on body temperature indices and feed intake.

	Day									SEM	*P*		
	P1*	1	2	3	4	5	6	7	8		Trt^1^	Day	TXD^2^
Tr^3^, °C													
PFTN^4^	39.32	39.08^c^	38.95^b^	38.92^b^	38.74^a^	38.82^ab^	38.83^ab^	38.82^ab^	38.82^ab^	0.10	<0.01	<0.01	<0.01
HS^5^	39.36	40.06^d^	40.73^g^	40.50^f^	40.31^e^	40.26^de^	40.29^de^	40.27^de^	40.26^de^	0.12			
RR, bpm^6^													
PFTN	40	34^b^	23^a^	21^a^	21^a^	19^a^	22^a^	24^a^	22^a^	4	<0.01	<0.01	<0.01
HS	32	88^c^	118^f^	98^de^	100^de^	96^cd^	105^e^	98^de^	92^de^	5			
FI^7^, kg													
PFTN	2.01	1.20^x^	1.16^x^	1.20^x^	1.15^x^	1.19^x^	1.18^x^	1.55^y^	1.23^x^	0.05	0.75	<0.01	0.99
HS	1.97	1.19^x^	1.14^x^	1.19^x^	1.14^x^	1.18^x^	1.16^x^	1.53^y^	1.21^x^	0.06			

1 Treatment.

2 Treatment by day interaction.

3 Rectal temperature.

4 Pair-fed thermoneutral.

5 Heat stress.

6 Respiration rate, breaths per minute.

7 Feed intake.

8 *Represents period 1 values that were statistically used as covariate.

^a–f^Means with different superscript differ (*P *≤* *0.05).

^x,y^Days with different superscript differ (*P *≤* *0.05).

**Figure 1:**
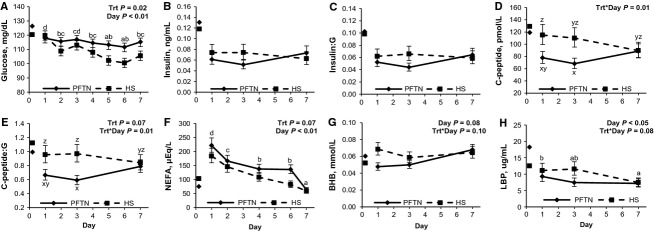
Effects of *ad libitum* feed intake in constant heat stress conditions (HS; 32°C) and pair-feeding in thermoneutral conditions (PFTN; 20°C) on temporal changes in plasma (A) glucose, (B) insulin, (C) insulin to glucose ratio (Insulin:G) (D) C-peptide, (E) C-peptide to glucose ratio (C-peptide:G), (F) nonesterified fatty acids (NEFA), (G) *β*-hydroxybutyrate (BHB), and (H) lipopolysaccharide-binding protein (LBP). Values on day 0 represent period 1 average that was statistically used as covariate. ^a-d^Means with different superscript differ (*P *≤* *0.05). ^x-^^z^Days with different superscript differ (*P *≤* *0.05).

**Figure 2:**
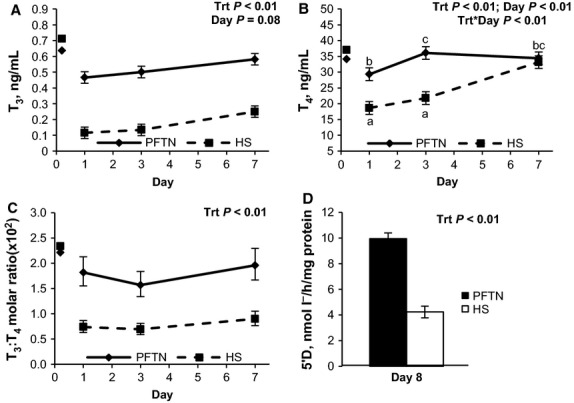
Effects of *ad libitum* feed intake in constant heat stress conditions (HS; 32°C) and pair-feeding in thermoneutral conditions (PFTN; 20°C) on temporal changes in plasma (A) T_3_, (B) T_4_, (C) T_3_ to T_4_ ratio (T_3_:T_4_); and (D) hepatic 5’-deiodinase type I activity (5'D) after 8 days of environmental treatment. Values on day 0 represent period 1 average that was statistically used as covariate. ^a–c^Means with different superscript differ (*P *≤* *0.05).

During period 2, basal glucose concentrations decreased progressively with time (*P *<* *0.01) in both treatments; however, HS pigs became overall more hypoglycemic (6%, *P *=* *0.02) than PFTN pigs (Fig.1A). No treatment differences were detected on basal plasma insulin (Fig.1B) or the insulin to glucose ratio (Fig.1C), but there was a treatment by day interaction on plasma C-peptide and C-peptide to glucose ratio (*P *=* *0.01) as they were increased on d 1 (48 and 44%, respectively) and 3 (61 and 64%, respectively) in HS pigs compared to PFTN controls, but no treatment differences were detected on d 7 (Fig.1D and E). During period 2, basal plasma NEFA acutely increased on d 1 and progressively decreased thereafter (*P *<* *0.01) in both treatments, but overall HS pigs tended to have reduced basal NEFA (20%, *P *=* *0.07) than PFTN pigs (Fig.1F). There was a tendency for a treatment by day interaction on plasma BHB (*P *=* *0.10), as HS pigs had increased BHB levels on d 1 (44%) compared to PFTN pigs, but similar BHB concentrations thereafter (Fig.1G). Circulating LBP decreased with time (*P *=* *0.05) and there was a tendency for a treatment by day interaction (*P *=* *0.08), as HS pigs had numerically increased LBP concentrations on d 1 (20%) and d 3 (56%) compared with PFTN controls, but plasma LBP did not differ between treatments on d 7 (Fig.1H). At the initiation of period 2, plasma T_3_ concentrations decreased, but progressively increased over time (*P *<* *0.01) for both treatments (Fig.2A). However, the d 1 decrease was more severe in HS pigs (68%) and this difference was maintained through the end of period 2 (*P *<* *0.01; Fig.2A). There was a treatment by day interaction on plasma T_4_ (*P *<* *0.01) as HS pigs had decreased concentrations compared to PFTN pigs on d 1 and 3 (36 and 40%, respectively), but circulating T_4_ did not differ between treatments on d 7 (Fig.2B). Overall, HS pigs had decreased T_3_ to T_4_ ratio (56%, *P *=* *0.01) compared to PFTN controls (Fig.2C). After 8 days of environmental treatment, hepatic 5′D activity was reduced (58%; *P *<* *0.01) in HS pigs compared to PFTN controls (Fig.2D).

During period 2 there was a treatment by day interaction on the glucose response to the GTT (*P *<* *0.01) as HS pigs had an increased glucose AUC (15%) on d 1, but not on d 6 (Table2). There was a treatment by day interaction on the glucose delta in response to the GTT (*P *=* *0.04), as it did not change from day 1 to 6 in PFTN pigs but decreased (10%) in HS pigs (Table2). Glucose disappearance following glucose administration tended to increase (13%; *P *=* *0.06) from day 1 to 6, however, overall, it tended to be decreased (14%; *P *=* *0.07) in HS pigs compared to PFTN counterparts (Table2). There was a tendency for a treatment by day interaction on the insulin response to the GTT (*P *=* *0.06) as HS pigs had a decreased (30%) insulin AUC on days 1 compared to PFTN pigs, but the insulin response was similar between treatments on d 6 (Table2). Overall, the insulin delta in response to the GTT tended to be reduced (16%, *P *=* *0.06) in HS pigs compared to PFTN controls (Table2). There was a treatment by day interaction on the insulinogenic index (*P *<* *0.01) as it was decreased (45%) in HS pigs on day 1 compared to PFTN controls, but did not differ between treatments on d 6 (Table2).

**Table 2:** Effects of pair-feeding or heat stress on the response to metabolic challenges.

Parameter	P^1^1*		Day 1–2 of P2		Day 6–7 of P2		SEM	*P*		
	PFTN^2^	HS^3^	PFTN	HS	PFTN	HS		Trt^4^	Day	TXD^5^
Glucose tolerance test										
Glucose AUC^6^, mg/dL·min	1991	1712	2257^a^	2595^b^	2273^a^	2189^a^	97	0.32	0.01	0.01
Glucose delta, mg/dL	172.6	171.4	186.1^ab^	198.3^b^	190.6^ab^	178.6^a^	7.5	0.99	0.18	0.04
Glucose disappearance, mg/dL/min	8.50	8.98	7.54	6.69	8.61	7.44	0.48	0.07	0.06	0.72
Insulin AUC, ng/mL·min	9.06	7.52	8.82	6.16	6.75	6.13	0.53	0.02	0.06	0.06
Insulin delta, ng/mL	0.58	0.54	0.47	0.39	0.43	0.36	0.03	0.06	0.11	0.89
Insulinogenic index, AU	5.20	4.85	4.53^b^	2.50^a^	3.68^a^	3.03^a^	0.43	0.04	0.47	0.01
Epinephrine challenge										
NEFA AUC, *μ*Eq/L·min	812	1260	1717	1309	1332	381	198	0.01	<0.01	0.17
Glucose AUC, mg/dL·min	519	433	620	580	689	685	77	0.80	0.23	0.80

1 Period.

2 Pair-fed thermoneutral.

3 Heat stress.

4 Treatment.

5 Treatment by day interaction.

6 Area under the curve.

7 *Represents period 1 values that were statistically used as covariate.

^a,b^Means with different superscript differ (*P *≤* *0.05).

During period 2, the NEFA AUC in response to the EC decreased (43%; *P *<* *0.01) from days 2 to 7, and overall it was reduced (71%; *P *=* *0.01) in HS pigs compared to PFTN controls (Table2). In contrast, the glucose response to the EC did not differ (*P *>* *0.10) between treatments or change with time (Table2).

**Figure 3:**
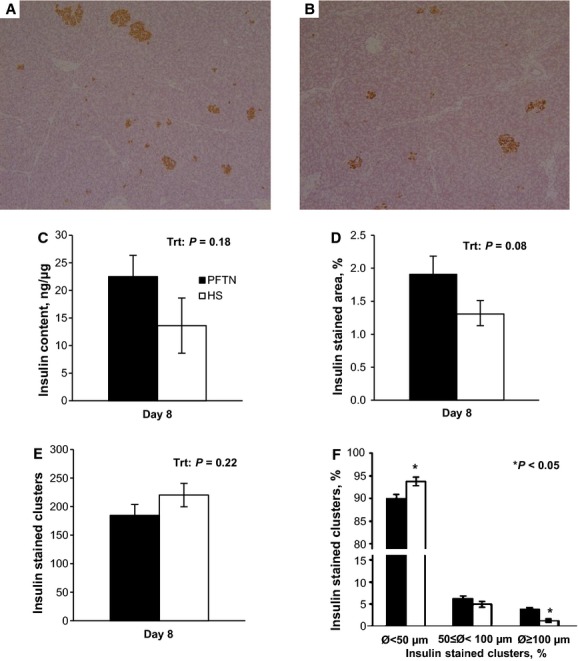
Effects of 8 days of *ad libitum* feed intake in constant heat stress conditions (HS; 32°C) and pair-feeding in thermoneutral conditions (PFTN; 20°C) pancreatic insulin content. (A) Representative picture of PFTN pancreas; (B) representative picture of PFTN pancreas; (C) insulin content; (D) insulin-stained area; (E) number of insulin-stained clusters; (F) insulin-stained clusters size distribution. *Represents differences with PFTN controls (*P *≤* *0.05).

After 8 days of environmental exposure, pancreatic insulin content did not differ (*P *>* *0.17) between treatments (Fig.3C). Based on the immunohistochemistry data, HS tended to decrease pancreatic insulin-stained area (32%; *P *=* *0.08) compared to PFTN conditions (Fig.3A, B, D). In addition, there were no treatment differences detected in the quantity of insulin-positive cell clusters (Fig.3E). However, when classified by size, HS decreased (66%; *P *<* *0.01) the percentage of larger clusters (diameter >100 *μ*m), whereas increasing (4%; *P *≤* *0.01) the percentage of smaller clusters (diameter <50 *μ*m; Fig.3F).

After 8 days of environmental treatment, HS increased or tended to increase transcript abundance for heat-shock proteins compared to PFTN conditions: *HSPA4* (2.43; *P *=* *0.08), *HSPB8* (2.51; *P *=* *0.10), and *HSPE1* (2.57; *P *=* *0.09), in AT; *HSP90AA1* (2.10; *P *<* *0.01), *HSPA4* (1.52; *P *=* *0.05), *HSPCB* (1.72; *P *=* *0.02), and *HSPE1* (2.38; *P *=* *0.02), in LD; and *HSPE1* (1.62; *P *=* *0.08), in liver (Tables3–5). In AT, transcripts for the following genes were or tended to be less abundant in HS compared to PFTN pigs: *ATGL* (−1.56; *P *=* *0.07), *INSR* (−1.46; *P *=* *0.07), *MIF* (−1.60; *P *=* *0.10), *PDAP1* (−1.73; *P *=* *0.07), *PRKAG1* (−1.89; *P *=* *0.05), and *ZSCAN29* (−1.77; *P *=* *0.05; Table3). In LD, HS conditions increased transcript abundance for *CD14* (1.68; *P *=* *0.01), and reduced or tended to reduce it for *CKMT2* (−2.03; *P *=* *0.10), *IDH2* (−1.77; *P *=* *0.06), *NDUFS7* (−2.01; *P *=* *0.06), *PDK4* (−2.53; *P *=* *0.05), and *SLC16A5* (−2.60; *P *=* *0.03); compared to PFTN conditions (Table4). In liver, mRNA transcripts for *FASN* (2.62; *P *<* *0.01), *KCTD6* (1.36; *P *=* *0.08), and *RBM7* (1.31; *P *=* *0.06) were or tended to be more abundant; whereas, *NDUFB7* (−1.28; *P *=* *0.05) and *NDUFS7* (−1.29; *P *=* *0.10) were or tended to be less abundant in HS pigs compared to PFTN controls (Table5).

**Table 3:** Effects of 8 days of pair-feeding or heat stress on gene expression in adipose tissue.

Gene	Description	Primers, 5′–3′	Trt, ΔΔ*C*_t_^1^		SEM	Fdiff^4^	*P*
			PFTN^2^	HS^3^			Trt^5^
ADRBK1	Adrenergic, beta, receptor kinase 1	F:GCGTCATGCAGAAGTACCTG^6^	−1.17	−0.53	0.28	−1.56	0.13
		R:GCTTCAGGCAGAAGTCTCGG^7^					
ATGL (PNPLA2)	Adipose triglyceride lipase (Patatin-like phospholipase domain containing 2)	F:GTGGCCACGGCCCTGGTTAC	−0.43	0.21	0.23	−1.56	0.07
		R:CGGTTCTTGGGCCCACTGCA					
ATP5J2	ATP synthase, H+ transporting, mitochondrial Fo complex, subunit F2	F:AGATGACGTCAGTTGTACCGC	0.69	0.28	0.30	1.33	0.35
		R:ATGCCCGAAGGGGTGAAATC					
CAPN1	Calpain 1, (mu/L) large subunit	F:GAGCTGTTCTCAAACCCCCA	−0.95	−0.50	0.22	−1.37	0.16
		R:GGGTGTCGTTGAGGGTAAGG					
CD14	CD14 molecule	F:CCTTGCAAGATCCTTCCGGT	0.10	−0.73	0.46	1.78	0.23
		R:TTGGCAGACTTTGGGGGTTT					
CIRBP	Cold inducible RNA-binding protein	F:GAGTCAGGGTGGCAGCTATG	0.70	0.12	0.31	1.49	0.21
		R:ACCCTTCTGAGTTGCACTGG					
FADS1	Fatty acid desaturase 1	F:CGTGATTGACCGGAAGGTGT	−2.26	−2.16	0.34	−1.07	0.83
		R:ACAAGGCCCTGGTTGATGTG					
FASN	Fatty acid synthase	F:CTCATCGGCGGTGTGGACAT	0.69	0.90	0.37	−1.16	0.69
		R:CATCGTGTTCGCCTGCTTGG					
G6PD	Glucose-6-phosphate dehydrogenase	F:GCGATGCTTTCCATCAGTCG	1.06	1.62	0.36	−1.47	0.29
		R:GCGTAGCCCACGATGTATGT					
GSK3B	Glycogen synthase kinase 3 beta	F:CGAGACACACCTGCACTCTT	−0.62	−0.18	0.29	−1.36	0.30
		R:TGACGCAGAAGCGGTGTTAT					
HSF1	Heat-shock transcription factor 1	F:TTCAAGCACAGCAACATGGC	−0.51	−0.19	0.26	−1.25	0.40
		R:CACGCTGGTCACTTTCCTCT					
HSF2	Heat-shock transcription factor 2	F:AGGCCAGGATGACTTGTTGG	−0.64	−1.12	0.41	1.39	0.43
		R:ACACCTCCTTCCAAAGGGAC					
HSP90AA1	Heat-shock protein 90 kDa alpha (cytosolic), class A member 1	F:ATCGCCCAGTTGATGTCGTT	0.21	−0.73	0.41	1.92	0.13
		R:TATCGTGAGGGTCCGGTCTT					
HSPA2	Heat-shock 70 kDa protein 2	F:TGAGAGTTTCCAGAAGGCGG	0.02	−0.92	0.55	1.92	0.25
		R:AAGACGAGCAAGCGACGTTA					
HSPA4	Heat-shock 70 kDa protein 4	F:CGCTTCGCAGTGTTTTGGAA	0.05	−1.23	0.48	2.43	0.08
		R:TGCACAGCCTCGAGTAACAG					
HSPB8	Heat-shock 22 kDa protein 8	F:GATGGCTACGTGGAGGTGTC	−0.09	−1.42	0.53	2.51	0.10
		R:GGGGAAAGCGAGGCAAATAC					
HSPCB	Heat-shock protein 90 kDa alpha (cytosolic), class B member 1	F:CCGTTCTCTTGAGTCACCCC	0.19	0.01	0.27	1.13	0.66
		R:GAGACATGAGCTGGGCGATT					
HSPE1	Heat-shock 10 kDa protein 1 (chaperonin 10)	F:AGCTGTTGGATCAGGCTCTAAA	1.76	0.40	0.52	2.57	0.09
		R:TGTGATGCCATTAGACAGTGAC					
IDH2	Isocitrate dehydrogenase 2 (NADP+), mitochondrial	F:CGACCAGAGGATCAAGGTGG	0.06	0.18	0.16	−1.09	0.59
		R:GGGAGCCCCAGGTCAAAATA					
INSR	Insulin receptor	F:CAGCCTGCGAGAGCGGATCG	−0.97	−0.42	0.19	−1.46	0.07
		R:TGAGAATAACCCCGGCCGCC					
LDHA	Lactate dehydrogenase A	F:AAGGAACACTGGAAAGCGG	1.10	0.56	0.47	1.45	0.44
		R:CATGGTGGAAATCGGATGCAC					
MAPK14	Mitogen-activated protein kinase 14	F:CAGGGGCTGAGCTTTTGAAG	−0.07	−0.39	0.24	1.25	0.35
		R:GCAAGTCAACAGCCAAGGGA					
MDH2	Malate dehydrogenase 2, NAD (mitochondrial)	F:TTCTTGCTGCCAGCTCGTTT	−0.05	0.46	0.41	−1.42	0.39
		R:AGCACAGCTACCTTGGCATT					
MIF	Macrophage migration inhibitory factor (glycosylation-inhibiting factor)	F:ATCAGCCCGGACAGGATCTA	0.02	0.70	0.27	−1.60	0.10
		R:GCCGAGAGCAAAGGAGTCTT					
MKNK2	MAP kinase interacting serine/threonine kinase 2	F:AAGAAGAAGAGGTGCCGAGC	−0.65	−0.10	0.25	−1.46	0.15
		R:CACCTCCCGGAAAACCCTAC					
NDUFB7	NADH dehydrogenase (ubiquinone) 1 beta subcomplex, 7, 18 kDa	F:CGCATGAAGGAGTTTGAGCG	−0.03	0.53	0.23	−1.47	0.12
		R:GGGGCCTGGAGGCTTTTATT					
NDUFS7	NADH dehydrogenase (ubiquinone) Fe-S protein 7, 20 kDa	F:TGCCAGAGCCTCGTTATGTT	0.13	0.42	0.23	−1.22	0.40
		R:CAGTCTCTTCTCCCGCTTGA					
NDUFV1	NADH dehydrogenase (ubiquinone) flavoprotein 1, 51 kDa	F:CGCGAGTATCTGTGCGTTTC	−0.17	0.32	0.22	−1.40	0.13
		R:TTCAGTCTCCAGTCATGGCG					
PC	Pyruvate carboxylase	F:AAGCCCTGGCCATAAGTGAC	0.30	0.48	0.24	−1.13	0.60
		R:CCCAATCTGGCCCTTCACAT					
PDAP1	PDGFA-associated protein 1	F:GTGGAAGGGCTCATCGACAT	0.61	1.40	0.28	−1.73	0.07
		R:GCTCTGTCTTCCCCGCTAAA					
PDCD7	Programmed cell death 7	F:TCTTACAGCCTTTCCGGCAG	−1.17	−1.22	0.37	1.04	0.92
		R:TTAATGGCGGTTGCCCAGAT					
PEMT	Phosphatidylethanolamine N-methyltransferase	F:ATGGAGCGCGTGTTTGACTA	0.32	0.40	0.27	−1.06	0.84
		R:TCCTGGGATCTCGTTCTCGT					
PLIN	Perilipin	F:CAACAAGGGCCTGACTTTGC	−1.63	−0.44	0.71	−2.28	0.26
		R:ATTGCATACAGACGCCACCA					
PRKAG1	Protein kinase, AMP-activated, gamma 1 noncatalytic subunit	F:GCATCCTCAAGACACCCCAG	−0.59	0.33	0.30	−1.89	0.05
		R:GCAGCTCGGACACCATTAGT					
PRLR	Prolactin receptor	F:ACAGTCACCTCCGGGAAAAC	−1.15	−0.92	0.63	−1.17	0.79
		R:TAGGGCCGCCAGTTTTGTAG					
RBM7	RNA-binding motif protein 7	F:AGCAGGTACGAAAGAACGGT	−0.13	−0.72	0.31	1.51	0.21
		R:GGGACTGAACTGATGGCGAA					
UBE2B	Ubiquitin-conjugating enzyme E2B	F:GGTGACCCACAGTGATTCGG	−1.03	−1.48	1.42	1.37	0.81
		R:AGGTGGGTCCTCTTGCAATC					
UBE2G1	Ubiquitin-conjugating enzyme E2G 1	F:TCGCCCGCTGTGTAAGAAAA	0.92	0.19	0.70	1.66	0.47
		R:CAGTTCCAGCCAGTGTTTGC					
ZAP70	Zeta-chain (TCR)-associated protein kinase 70 kDa	F:CAACTTTGGCTCTGTTCGCC	0.35	1.54	0.57	−2.28	0.19
		R:CACGTTGCTGACAGGGATCT					
ZSCAN29	Zinc finger and SCAN domain containing 29	F:CCCACAGGAGAAGCTCAGAC	−1.13	−0.31	0.26	−1.77	0.05
		R:CCCAGTGATCCTGTTCCACC					

1 Delta delta *C*_t_.

2 Pair-fed thermoneutral.

3 Heat stress.

4 Fold difference: positive/negative values indicate increased/decreased transcript abundance in HS pigs compared to PFTN controls.

5 Treatment.

6 Forward.

7 Reverse.

**Table 4:** Effects of 8 days of pair-feeding or heat stress on gene expression in skeletal muscle (*Longissimus dorsi*).

Gene	Description	Primers, 5′–3′	Trt, ΔΔ*C*_t_^1^		SEM	Fdiff^4^	*P*
			PFTN^2^	HS^3^			Trt^5^
ADRBK1	Adrenergic, beta, receptor kinase 1	F:GCGTCATGCAGAAGTACCTG^6^	1.28	1.37	0.24	−1.06	0.80
		R:GCTTCAGGCAGAAGTCTCGG^7^					
ATP5J2	ATP synthase, H+ transporting, mitochondrial Fo complex, subunit F2	F:GATGACGTCAGTTGTACCGC	0.25	0.51	0.35	−1.20	0.60
		R:AATGCCCGAAGGGGTGAAAT					
CAPN1	Calpain 1, (mu/L) large subunit	F:TGCACCGAGTAGTTCCACAC	0.67	0.70	0.38	−1.02	0.96
		R:AACTCATTGCCTTGGGCAGA					
CD14	CD14 molecule	F:AACCCCCAAAGTCTGCCAAA	0.83	0.08	0.17	1.68	0.01
		R:AAGGTCCTCAAAGCCTCTGC					
CIRBP	Cold inducible RNA-binding protein	F:GAGTCAGGGTGGCAGCTATG	0.27	0.43	0.30	−1.12	0.71
		R:ACCCTTCTGAGTTGCACTGG					
CKMT2	Creatine kinase, mitochondrial 2 (sarcomeric)	F:CTAACTGGCCGCAATGCTTC	0.87	1.89	0.41	−2.03	0.10
		R:GCAGTTGTTGTGCTTGCGTA					
CPT2	Carnitine palmitoyltransferase 2	F:TCAGGCGGTAAGATGTGGAA	−0.35	−0.03	0.29	−1.25	0.45
		R:ACCATTACCTGAAGAGCCCG					
DDB1	Damage-specific DNA-binding protein 1, 127 kDa	F:AAGCGCCTACCATCTGCTTT	1.10	1.32	0.23	−1.16	0.51
		R:TCTGGCACTGCAATCACCAT					
FAR1	Fatty acyl CoA reductase 1	F:TCCAACAATGCCCTTGCAGA	0.25	0.18	0.25	1.05	0.83
		R:GGGCCTTATGGCTTACAGCA					
G6PD	Glucose-6-phosphate dehydrogenase	F:GCGATGCTTTCCATCAGTCG	1.04	1.25	0.16	−1.16	0.35
		R:GCGTAGCCCACGATGTATGT					
HIF1a	Hypoxia inducible factor 1, alpha subunit	F:ATCTCGGGCACAGATTCGC	0.10	−0.19	0.21	1.22	0.34
		R:TCCTCACACGCAAATAGCTGA					
HSF1	Heat-shock transcription factor 1	F:TTCAAGCACAGCAACATGGC	1.99	1.94	0.30	1.04	0.90
		R:CACGCTGGTCACTTTCCTCT					
HSF2	Heat-shock transcription factor 2	F:AGGCCAGGATGACTTGTTGG	0.46	0.35	0.24	1.08	0.76
		R:ACACCTCCTTCCAAAGGGAC					
HSP90AA1	Heat-shock protein 90 kDa alpha (cytosolic), class A member 1	F:ATCGCCCAGTTGATGTCGTT	1.02	−0.05	0.18	2.10	<0.01
		R:TATCGTGAGGGTCCGGTCTT					
HSPA2	Heat-shock 70 kDa protein 2	F:TGAGAGTTTCCAGAAGGCGG	−0.06	−0.01	0.53	−1.04	0.94
		R:AAGACGAGCAAGCGACGTTA					
HSPA4	Heat-shock 70 kDa protein 4	F:AAAGATTCCATGGCCGAGCA	0.88	0.28	0.19	1.52	0.05
		R:GCCGTCTCCTTCAGTTTGGA					
HSPB8	Heat-shock 22 kDa protein 8	F:GATGGCTACGTGGAGGTGTC	1.33	0.91	0.22	1.34	0.20
		R:GGGGAAAGCGAGGCAAATAC					
HSPCB	Heat-shock protein 90 kDa alpha (cytosolic), class B member 1	F:AGCCGTTCTCTTGAGTCACC	0.79	0.01	0.20	1.72	0.02
		R:TGCCTGGAAGGCAAAAGTCT					
HSPE1	Heat-shock 10 kDa protein 1 (chaperonin 10)	F:TTGCAAGCAACCGTAGTAGC	0.17	−1.08	0.34	2.38	0.02
		R:ACAGTGACTTGTTTCAGTCTACG					
IDH2	Isocitrate dehydrogenase 2 (NADP+), mitochondrial	F:CGACCAGAGGATCAAGGTGG	0.73	1.55	0.28	−1.77	0.06
		R:GGGAGCCCCAGGTCAAAATA					
KCTD6	Potassium channel tetramerization domain containing 6	F:TGGATAATGGAGACTGGGGCT	0.75	0.90	0.34	−1.11	0.76
		R:AGCATGGAATCCGGGTAACG					
LDHA	Lactate dehydrogenase A	F:TTGTTGGGGTTGGTGCTGTT	1.14	1.83	0.43	−1.61	0.28
		R:TGGGGTCCTAAGGAAAAGGC					
MAPK14	Mitogen-activated protein kinase 14	F:AAGACTCGTTGGAACCCCAG	1.41	1.14	0.24	1.21	0.43
		R:TCCAGCAAGTCAACAGCCAA					
MDH2	Malate dehydrogenase 2, NAD (mitochondrial)	F:TTTCTTGCTGCCAGCTCGTT	1.46	2.37	0.41	−1.88	0.14
		R:GCACAGCTACCTTGGCATTG					
MEF2A	Myocyte enhancer factor 2A	F:AAGGAACACAGAGGGTGCG	0.64	0.72	0.24	−1.06	0.81
		R:AAAGCATTAGGGCTGGTCACA					
MIF	Macrophage migration inhibitory factor (glycosylation-inhibiting factor)	F:ATCAGCCCGGACAGGATCTA	−0.07	−0.23	0.19	1.12	0.58
		R:GCCGAGAGCAAAGGAGTCTT					
MKNK2	MAP kinase interacting serine/threonine kinase 2	F:AAGAAGAAGAGGTGCCGAGC	1.13	1.40	0.28	−1.21	0.51
		R:CACCTCCCGGAAAACCCTAC					
MYOD1	Myogenic differentiation 1	F:CTACAGCGGTGACTCAGACG	1.24	1.52	0.33	−1.21	0.57
		R:AATAGGTGCCGTCGTAGCAG					
NDUFA6	NADH dehydrogenase (ubiquinone) 1 alpha subcomplex, 6, 14 kDa	F:CAGAGCCTTGCATGTCGGTA	0.59	1.08	0.32	−1.40	0.29
		R:AGCTAACCAATCCTGGTGGC					
NDUFB7	NADH dehydrogenase (ubiquinone) 1 beta subcomplex, 7, 18 kDa	F:CGCATGAAGGAGTTTGAGCG	0.10	0.47	0.34	−1.29	0.45
		R:GGGGCCTGGAGGCTTTTATT					
NDUFS7	NADH dehydrogenase (ubiquinone) Fe-S protein 7, 20 kDa	F:TGCCAGAGCCTCGTTATGTT	0.28	1.29	0.36	−2.01	0.06
		R:CAGTCTCTTCTCCCGCTTGA					
NDUFV1	NADH dehydrogenase (ubiquinone) flavoprotein 1, 51 kDa	F:CGCGAGTATCTGTGCGTTTC	1.21	1.88	0.32	−1.59	0.16
		R:TTCAGTCTCCAGTCATGGCG					
PC	Pyruvate carboxylase	F:AAGCCCTGGCCATAAGTGAC	2.65	3.08	0.35	−1.35	0.39
		R:CCCAATCTGGCCCTTCACAT					
PDAP1	PDGFA-associated protein 1	F:GTGGAAGGGCTCATCGACAT	0.56	0.51	0.28	1.04	0.91
		R:GCTCTGTCTTCCCCGCTAAA					
PDK4	Pyruvate dehydrogenase kinase, isozyme 4	F:AAGCCACATTGGCAGCATTG	−0.78	0.56	0.44	−2.53	0.05
		R:GGTGTTCGACTGTAGCCCTC					
PEMT	Phosphatidylethanolamine N-methyltransferase	F:ATGGAGCGCGTGTTTGACTA	2.55	3.11	0.33	−1.47	0.26
		R:TCCTGGGATCTCGTTCTCGT					
PRKAG1	Protein kinase, AMP-activated, gamma 1 noncatalytic subunit	F:GCATCCTCAAGACACCCCAG	0.59	0.85	0.25	−1.20	0.48
		R:GCAGCTCGGACACCATTAGT					
RASA1	RAS p21 protein activator (GTPase activating protein) 1	F:GGGAAGGGCAAAACCCTGTA	0.11	0.05	0.16	1.04	0.81
		R:GCTCAATTGGCAGCGCATAA					
RBM7	RNA-binding motif protein 7	F:GCAGGTACGAAAGAACGGTG	0.27	0.16	0.24	1.08	0.76
		R:ATGGGACTGAACTGATGGCG					
SLC16A5	Solute carrier family 16, member 5	F:TCGGCATCTTCTTCACCGAA	1.23	2.61	0.40	−2.60	0.03
		R:GATGAATCCTGCCGTGAGGT					
SLC25A27	Solute carrier family 25, member 27	F:AAGCTCTGGCAAGGAGTGAC	0.76	0.07	0.29	1.61	0.12
		R:CACGAAATCGCAACGGCTTT					
UBE2G1	Ubiquitin-conjugating enzyme E2G 1	F:TCGCCCGCTGTGTAAGAAAA	0.26	0.36	0.35	−1.07	0.83
		R:CAGTTCCAGCCAGTGTTTGC					
ZAP70	Zeta-chain (TCR)-associated protein kinase 70 kDa	F:CAACTTTGGCTCTGTTCGCCx	−0.05	0.40	0.33	−1.37	0.35
		R:CAGTTCCAGCCAGTGTTTGC					
ZSCAN29	Zinc finger and SCAN domain containing 29	F:CCCACAGGAGAAGCTCAGAC	1.26	1.06	0.16	1.15	0.39
		R:CCCAGTGATCCTGTTCCACC					

1 Delta delta *C*_t_.

2 Pair-fed thermoneutral.

3 Heat stress.

4 Fold difference: positive/negative values indicate increased/decreased transcript abundance in HS pigs compared to PFTN controls.

5 Treatment.

6 Forward.

7 Reverse.

**Table 5:** Effects of 8 days of pair-feeding or heat stress on gene expression in liver.

Gene	Description	Primers, 5′–3′	Trt, ΔΔ*C*_t_^1^		SEM	Fdiff^4^	*P*
			PFTN^2^	HS^3^			Trt^5^
ADRBK1	Adrenergic, beta, receptor kinase 1	F:GCGTCATGCAGAAGTACCTG^6^	0.18	0.02	0.18	1.12	0.54
		R:GCTTCAGGCAGAAGTCTCGG^7^					
ATP5J2	ATP synthase, H+ transporting, mitochondrial Fo complex, subu F2	F:GATGACGTCAGTTGTACCGC	0.08	0.34	0.12	−1.20	0.14
		R:AATGCCCGAAGGGGTGAAAT					
CAPN1	Calpain 1, (mu/L) large subunit	F:TGCACCGAGTAGTTCCACAC	0.52	0.70	0.59	−1.13	0.17
		R:AACTCATTGCCTTGGGCAGA					
CCRN4L	CCR4 carbon catabolite repression 4-like (S. cerevisiae)	F:CCCGCTTCCAGAGGGATTTT	0.94	1.49	0.34	−1.46	0.27
		R:GGGCTGGTAGGCTAGGATCT					
CD14	CD14 molecule	F:AACCCCCAAAGTCTGCCAAA	−0.32	0.08	0.26	−1.32	0.28
		R:AAGGTCCTCAAAGCCTCTGC					
CIRBP	Cold inducible RNA-binding protein	F:GAGTCAGGGTGGCAGCTATG	0.13	0.18	0.13	−1.04	0.81
		R:ACCCTTCTGAGTTGCACTGG					
FADS2	Fatty acid desaturase 2	F:GCGCAGATGCCTACCTTT	0.48	0.65	0.25	−1.13	0.64
		R:CTAAATCAAGGTCGCGGTGG					
FASN	Fatty acid synthase	F:TCATCGGCGGTGTGGACAT	2.56	1.17	0.24	2.62	<0.01
		R:CCATCGTGTTCGCCTGCTTG					
G6PD	Glucose-6-phosphate dehydrogenase	F:GCGATGCTTTCCATCAGTCG	1.56	0.99	0.46	1.48	0.39
		R:GCGTAGCCCACGATGTATGT					
GCK	Glucokinase (hexokinase 4)	F:CTGCCTTGGAAGCCTGTTGG	2.36	1.82	0.64	1.45	0.56
		R:ATCTCCTTCTGCATCCGCCT					
HIF1a	Hypoxia inducible factor 1, alpha subunit	F:ATCTCGGGCACAGATTCGC	1.02	0.42	0.46	1.52	0.37
		R:TCCTCACACGCAAATAGCTGA					
HSF1	Heat-shock transcription factor 1	F:TTCAAGCACAGCAACATGGC	0.14	0.14	0.20	1.00	1.00
		R:CACGCTGGTCACTTTCCTCT					
HSF2	Heat-shock transcription factor 2	F:AGGCCAGGATGACTTGTTGG	0.14	−0.07	0.17	1.16	0.40
		R:ACACCTCCTTCCAAAGGGAC					
HSP90AA1	Heat-shock protein 90 kDa alpha (cytosolic), class A member 1	F:ATCGCCCAGTTGATGTCGTT	0.53	0.38	0.13	1.11	0.45
		R:TATCGTGAGGGTCCGGTCTT					
HSPA2	Heat-shock 70 kDa protein 2	F:TGAGAGTTTCCAGAAGGCGG	−0.46	−0.62	0.52	1.12	0.83
		R:AAGACGAGCAAGCGACGTTA					
HSPA4	Heat-shock 70 kDa protein 4	F:AAAGATTCCATGGCCGAGCA	0.59	0.26	0.14	1.26	0.12
		R:GCCGTCTCCTTCAGTTTGGA					
HSPB8	Heat-shock 22 kDa protein 8	F:GATGGCTACGTGGAGGTGTC	0.28	0.39	0.18	−1.08	0.69
		R:GGGGAAAGCGAGGCAAATAC					
HSPCB	Heat-shock protein 90 kDa alpha (cytosolic), class B member 1	F:AGCCGTTCTCTTGAGTCACC	−0.10	−0.18	0.24	1.06	0.81
		R:TGCCTGGAAGGCAAAAGTCT					
HSPE1	Heat-shock 10 kDa protein 1 (chaperonin 10)	F:TTGCAAGCAACCGTAGTAGC	1.11	0.41	0.26	1.62	0.08
		R:ACAGTGACTTGTTTCAGTCTACG					
IDH2	Isocitrate dehydrogenase 2 (NADP+), mitochondrial	F:CGACCAGAGGATCAAGGTGG	0.69	0.40	0.30	1.22	0.50
		R:GGGAGCCCCAGGTCAAAATA					
KCTD6	Potassium channel tetramerization domain containing 6	F:TGGATAATGGAGACTGGGGCT	0.53	0.09	0.16	1.36	0.08
		R:AGCATGGAATCCGGGTAACG					
LDHA	Lactate dehydrogenase A	F:TTGTTGGGGTTGGTGCTGTT	0.74	0.76	0.20	−1.01	0.96
		R:TGGGGTCCTAAGGAAAAGGC					
LIPG	Lipase, endothelial	F:CTGGTTCTGGTTCAAGCCCT	0.09	0.32	0.31	−1.17	0.60
		R:GATCAGACAGTGGTGGCCTT					
MAPK14	Mitogen-activated protein kinase 14	F:CAGGGGCTGAGCTTTTGAAG	0.84	0.34	0.29	1.41	0.25
		R:GCAAGTCAACAGCCAAGGGA					
MDH2	Malate dehydrogenase 2, NAD (mitochondrial)	F:TTTCTTGCTGCCAGCTCGTT	0.54	0.71	0.44	−1.13	0.80
		R:GCACAGCTACCTTGGCATTG					
MIF	Macrophage migration inhibitory factor (glycosylation-inhibiting factor)	F:ATCAGCCCGGACAGGATCTA	0.67	0.59	0.31	1.06	0.85
		R:GCCGAGAGCAAAGGAGTCTT					
MKNK2	MAP kinase interacting serine/threonine kinase 2	F:AAGAAGAAGAGGTGCCGAGC	0.73	0.38	0.23	1.27	0.29
		R:CACCTCCCGGAAAACCCTAC					
NDUFA6	NADH dehydrogenase (ubiquinone) 1 alpha subcomplex, 6, 14 kDa	F:CAGAGCCTTGCATGTCGGTA	−0.91	−1.02	0.15	1.08	0.60
		R:AGCTAACCAATCCTGGTGGC					
NDUFB7	NADH dehydrogenase (ubiquinone) 1 beta subcomplex, 7, 18 kDa	F:CGCATGAAGGAGTTTGAGCG	0.12	0.48	0.12	−1.28	0.05
		R:GGGGCCTGGAGGCTTTTATT					
NDUFS7	NADH dehydrogenase (ubiquinone) Fe-S protein 7, 20 kDa	F:TGCCAGAGCCTCGTTATGTT	0.02	0.39	0.16	−1.29	0.10
		R:CAGTCTCTTCTCCCGCTTGA					
NDUFV1	NADH dehydrogenase (ubiquinone) flavoprotein 1, 51 kDa	F:CGCGAGTATCTGTGCGTTTC	0.10	0.41	0.22	−1.24	0.32
		R:TTCAGTCTCCAGTCATGGCG					
PC	Pyruvate carboxylase	F:AAGCCCTGGCCATAAGTGAC	−0.38	−0.34	0.20	−1.03	0.87
		R:CCCAATCTGGCCCTTCACAT					
PDAP1	PDGFA-associated protein 1	F:GTGGAAGGGCTCATCGACAT	1.10	0.90	0.32	1.15	0.66
		R:GCTCTGTCTTCCCCGCTAAA					
PDK4	Pyruvate dehydrogenase kinase, isozyme 4	F:AAGCCACATTGGCAGCATTG	−0.28	−0.46	0.14	1.13	0.37
		R:GGTGTTCGACTGTAGCCCTC					
PEMT	Phosphatidylethanolamine N-methyltransferase	F:ATGGAGCGCGTGTTTGACTA	−0.57	−0.43	0.17	−1.10	0.56
		R:TCCTGGGATCTCGTTCTCGT					
POLD4	Polymerase (DNA-directed), delta 4, accessory subunit	F:GCTCTGCTGTGAAGTTTGGC	0.13	0.14	0.13	1.00	0.90
		R:AGCCTTTGGAAGGGTCATGG					
PRKAG1	Protein kinase, AMP-activated, gamma 1 noncatalytic subunit	F:GCATCCTCAAGACACCCCAG	0.38	0.05	0.28	1.26	0.43
		R:GCAGCTCGGACACCATTAGT					
RBM7	RNA-binding motif protein 7	F:AGCAGGTACGAAAGAACGGT	0.33	−0.06	0.13	1.31	0.06
		R:GGGACTGAACTGATGGCGAA					
SDHC	Succinate dehydrogenase complex, subunit. C, 15 kDa	F:CCGTGCCCATCTTAGTCCTC	0.59	0.55	0.21	1.03	0.89
		R:GGGGAGACAAAGGACGGTTT					
SLC16A5	Solute carrier family 16, member 5	F:TCGGCATCTTCTTCACCGAA	0.15	0.14	0.34	1.01	0.98
		R:GATGAATCCTGCCGTGAGGT					
SLC25A27	Solute carrier family 25, member 27	F:AAGCTCTGGCAAGGAGTGAC	−0.02	−0.23	0.19	1.16	0.44
		R:CACGAAATCGCAACGGCTTT					
UBE2G1	Ubiquitin-conjugating enzyme E2G 1	F:ACTCGCCTGCTAATGTGGAC	0.45	0.16	0.34	1.22	0.56
		R:GTGCAGGAAAAACAGTGCCA					
ZAP70	Zeta-chain (TCR)-associated protein kinase 70 kDa	F:CAACTTTGGCTCTGTTCGCC	−0.07	0.04	0.16	−1.08	0.63
		R:CACGTTGCTGACAGGGATCT					
ZSCAN29	Zinc finger and SCAN domain containing 29	F:CCCACAGGAGAAGCTCAGAC	−0.24	−0.43	0.21	1.14	0.54
		R:CCCAGTGATCCTGTTCCACC					

1 Delta delta *C*_t_.

2 Pair-fed thermoneutral.

3 Heat stress.

4 Fold difference: positive/negative values indicate increased/decreased transcript abundancein HS pigs compared to PFTN controls.

5 Treatment.

6 Forward.

7 Reverse.

## Discussion

Heat stress jeopardizes human health and compromises animal agriculture productivity. Despite advances in the understanding of heat-related illnesses, there is no treatment against specific aspects of their pathophysiology, and protocols are limited to generic cooling and hydration (Leon and Helwig 2010). Physically modifying the microenvironment with heat stress abatement strategies (shade, fans, evaporative cooling) has markedly enhanced agricultural productivity, but facility improvements have failed to completely prevent the loss in animal efficiency during the warm summer months (Stowell et al. 2009). Therefore, a better understanding of the biological consequences of HS is a prerequisite to developing treatment protocols and mitigation strategies targeting the physiological and metabolic ramifications of heat-related illnesses. The pig is recognized as an excellent biomedical model (Prather et al. 2013) and the highly conserved metabolic and physiological responses to a severe heat load amongst species (Baumgard and Rhoads 2013) make it an ideal model to further our appreciation of thermal biology.

Typically, during inadequate nutrient intake AT is mobilized and NEFA contribution to whole-body oxidation is markedly increased (Vernon 1992). However, in this study, despite a 40% reduction in FI, HS pigs tended to have reduced basal NEFA concentrations and this suggests that they did not mobilize as much AT as PFTN counterparts. Furthermore, the NEFA response to the EC was blunted in the HS pigs and this reduced sensitivity to lipolytic signals agrees with our ruminant data (Baumgard et al. 2011). The decrease in circulating markers of AT mobilization during HS is mechanistically supported by a tendency for a reduction in *ATGL* gene expression, a key enzyme of the lipolytic cascade (Zimmermann et al. 2004), as well as the significant reduction in the gene encoding the AMPK regulatory subunit (*PRKAG1*). Interestingly, recent work has implicated AMPK in the regulation of ATGL content and lipase activity (Gaidhu et al. 2012), and it is tempting to speculate that their simultaneous reduction in transcript abundance is mechanistically related. The reduced capacity for AT to contribute to systemic energetics despite an increase in energy requirements during HS is consistent with research in agricultural animals and biomedical models (Torlinska et al. 1987; Shwartz et al. 2009; Pearce et al. 2013a). Moreover, similar to our ruminant data (Baumgard et al. 2011), the glucose response to the EC, an indicator of hepatic glycogenolysis capacity, was similar between HS and PFTN pigs. These data indicate that AT becomes refractory to adrenergic signals, whereas the liver remains responsive during HS. The mechanism for the different responsiveness between the liver and AT is unknown, but a rationale for it could be the increased reliance on glucose as a whole-body fuel during HS like we and others have demonstrated [see review (Baumgard and Rhoads 2013)]. By remaining sensitive to catabolic signals, the liver can maintain its pivotal role as the glucose supplier to extrahepatic tissues. Acute HS (24 h) increased plasma BHB, which is somewhat perplexing as circulating BHB is normally positively correlated with NEFA levels (Masoro 1977). However, this is not unprecedented as Ronchi et al. (Ronchi et al. 1999) reported a similar discordant NEFA and BHB pattern in HS ruminants. The fact that circulating BHB increases without a concomitant increase in circulating NEFA might indicate that ketone body oxidation is decreased; thereby reduce their plasma clearance. Interestingly, HS increases circulating LPS (presumably from intestinal origin as described later in this discussion) and we have recently demonstrated that LPS exposure reduces skeletal myoblast ketone oxidation (R. P. Rhoads and L. H. Baumgard, unpublished observation). Nonetheless, the mechanism by which HS uncouples the relationship between circulating NEFA and BHB and its energetic consequences require further investigation.

Reasons why heat-stressed animals fail to mobilize AT despite being in a hypercatabolic condition might be related to changes in insulin homeostasis. Insulin is a potent antilipolytic signal and is frequently elevated in HS animals when compared with PFTN counterparts (Baumgard and Rhoads 2013). In contrast, we did not observe treatment differences in basal insulin; however, C-peptide, a coproduct of insulin's cleavage from proinsulin, was increased in HS pigs. C-peptide is thought to be a better indicator of pancreatic insulin secretion because it is produced at a 1:1 ratio to insulin and it avoids the confounding effects of hepatic insulin extraction (Wallace et al. 2004). Our observation of increased C-peptide suggests that HS-induced increase in circulating insulin is the result of enhanced pancreatic secretion rather than decreased systemic insulin clearance. Furthermore, increased insulin secretion in heat-stressed pigs was associated with pancreatic insulin depletion as HS decreased pancreatic insulin content, ostensibly (i.e., insulin-stained area was reduced and pancreatic insulin protein content was numerically decreased) due to a reduction in insulin-positive cluster size rather than cluster number. Interestingly, insulin receptor (*INSR*) gene expression at the adipose tissue level tended to be decreased in HS pigs, which is presumably an adaptive response to increased circulating insulin.

Exposing *β* cells to HS *in vitro* decreases insulin secretion (Kondo et al. 2012), which suggests that the heat-induced circulating insulin response we observe *in vivo* might be triggered by secondary signals rather than direct pancreatic hyperthermia. One example may be prolactin, which increases in response to HS in a variety of species and models (Alamer 2011), including humans (Iguchi et al. 2012) and our HS pig model (Sanz-Fernandez et al. 2012). Prolactin is thought to be involved in water homeostasis and the sweating response (Alamer 2011); however, its role in HS adaptation is not fully under-stood. Intriguingly, prolactin increases *β* cell proliferation and glucose-stimulated insulin secretion *in vivo* and *in vitro* (Brelje and Sorenson 1991; Hughes and Huang 2011). Thus, determining prolactin's role in HS metabolic adaptation is of obvious interest. An additional signal potentially involved in the insulin response during HS is LPS. Heat stress increases intestinal permeability to luminal content due to redistribution of blood flow from the viscera to the periphery in an attempt to maximize radiant heat dissipation (Lambert et al. 2002). The decrease in intestinal perfusion leads to mucosal hypoxia which compromises the intestinal barrier function (Hall et al. 1999). In agreement, we observe an increase in intestinal permeability and the subsequent increase in circulating LPS in our model (Pearce et al. 2013b; Sanz Fernandez et al. 2014), and an LPS challenge acutely increases circulating insulin both *in vivo* (Burdick Sanchez et al. 2013) and *in vitro* (Vives-Pi et al. 2003). Interestingly, in this study *CD14* gene expression in LD and circulating LBP, both key proteins in the recognition of LPS by Toll-like receptor 4 (Lu et al. 2008), were upregulated and numerically increased, respectively, in HS pigs. Furthermore, the partial loss of differences in metabolism between treatments by d 6 of environmental exposure might correspond to the temporal pattern of acclimation by the intestinal barrier. This hypothesis is supported by the lack of differences in LBP by the end of the experiment. Moreover, because we utilized jugular catheters we administered antibiotics throughout the length of the experiment to prevent infection. This experimental approach may have influenced the intestinal flora, mitigating the leakage of luminal proinflammatory molecules to the portal and systemic blood stream, and/or the immune response to them. Determining how antibiotics influence the metabolic and inflammatory response to HS is of academic and practical interest.

Contrary to our previous studies in ruminants (O'Brien et al. 2010; Wheelock et al. 2010), the insulin response to the GTT was decreased by HS. This might be due to species differences in insulin responsiveness as pigs are generally considered more insulin sensitive than ruminants (Brockman and Laarveld 1986), and therefore, changes in peripheral insulin sensitivity might be adequate for insulin to exert its effects on HS adaptation in swine. The effects of HS on whole-body insulin sensitivity is not clear as there is evidence that it either increases, does not change or actually decreases [see review (Baumgard and Rhoads 2013)]. In this study, the rate of glucose disposal following the GTT was decreased by HS and this suggests insulin resistance, but this is likely explained by a decreased insulin response to the GTT in HS pigs. Coupling the insulin response with the glucose disposal parameters (the insulinogenic index) suggests that HS pigs actually required less insulin to stimulate a similar quantity of systemic glucose uptake. The increased insulin sensitivity agrees with previous reports where thermal therapy improved insulin action in diabetic and obese rodents (Kokura et al. 2007; Gupte et al. 2009) and humans (Hooper 1999), and might be the result of heat-shock protein upregulation, as overexpression of HSP72 protects against obesity-induced insulin resistance and hsp coinducers improve insulin sensitivity (Chung et al. 2008). Consequently, both the increase in basal circulating insulin and the apparent enhanced insulin sensitivity seem to be critical for survival and adaptation to a heat load as diabetics are more susceptible to heat related-illness/death and insulin administration to diabetic rodents improves survivability to severe HS (Semenza et al. 1999; Niu et al. 2003).

Heat stress appears to markedly alter intracellular energetics, characterized by a decrease in ATP production via oxidative phosphorylation and an increase in energy production via aerobic glycolysis (Baumgard and Rhoads 2013) and this resembles the Warburg effect utilized by cancer cells (Kim and Dang 2006). Our molecular data agree with this tenet as the gene expression of the tricarboxylic acid cycle (*IDH2*) and electron transport chain enzymes (*NDUFB7, NDUFS7*), as well as mitochondrial creatine kinase (*CKMT2*; responsible for the transfer of high energy phosphate from the mitochondria to the cytosol) were or tended to be downregulated in LD and liver of HS compared to PFTN pigs. In contrast to our previous findings (Sanders et al. 2009; Won et al. 2012), LD pyruvate dehydrogenase kinase 4 (*PDK4*), which inactivates the pyruvate dehydrogenase complex and thus regulates glucose flux from glycolysis into the TCA cycle, was downregulated in HS pigs. Reasons for these dissimilar results may include differences in experimental design, as in the past we have compared HS animals to TN *ad libitum* controls instead of to PFTN ones, and feed restriction by itself increases PDK4 expression (Furuyama et al. 2003). Nevertheless, the pyruvate dehydrogenase complex is regulated by several PDK isozymes as well as pyruvate dehydrogenase phosphatases (Harris et al. 2002), so further research is required in order to establish its role in the cellular metabolic shift during HS.

The decreased AT mobilization and improved insulin sensitivity observed in the HS pigs might actually be the result of reduced energetic requirements. Traditionally, HS has been thought to increase whole-body energy expenditure due to employing heat dissipation mechanisms (panting, etc.; Fuquay 1981). Furthermore, despite the questionable applicability of the van't Hoff–Arrhenius equation (a formula stating that chemical reactions are temperature dependent and that reaction rates increase as temperature rises) to homeotherms, it is frequently touted as being partially responsible for the increase in maintenance costs of heat-stressed animals (see review: Baumgard and Rhoads 2013). Furthermore, the potential activation of the immune system as a consequence of the HS-induced increased intestinal permeability comes with a high energetic cost. In an apparent contrast, heat-stressed animals experience a marked decrease in thyroid hormones, which are typically correlated with whole-body energy expenditure and heat production [as reviewed in (Baumgard and Rhoads 2013)]. In our study, after only 24 h of HS exposure, both circulating T_3_ and T_4_ were sharply decreased, which suggests that in the early stages of HS the reduction in thyroid hormones is related to reduced thyroid gland activity. However, T_4_ progressively increased to baseline levels, whereas T_3_ and similarly T_3_:T_4_ ratio remained low throughout the experiment. This implies that as HS progresses thyroid gland acclimates, but the extrathyroidal T_4_ to T_3_ conversion (responsible for most of the circulating T_3_ in mammals (Kahl et al. 2002) remains reduced. In agreement, by d 8 of HS, hepatic 5′D activity was decreased in HS pigs compared to PFTN animals. Interestingly, thyroid hormones can activate lipolysis and NEFA utilization (Pucci et al. 2000) and thus the heat-induced reduction in adipose tissue mobilization might be the result of decreased thyroid hormones’ stimulation. Furthermore, hypothyroid humans have reduced sensitivity to adrenergic signals (Mullur et al. 2014), which may explain why HS pigs had a blunted NEFA response to epinephrine. The mechanism by which HS decreases thyroid parameters are not fully understood, but both cortisol and LPS have been shown to regulate aspects of the thyroid axis in different models (Kahl et al. 2000; Larson 2005) and both are increased in acute HS (Baumgard and Rhoads 2013). Determining the energetic and nutrient demand of heat-stressed animals would presumably provide clues on how to better treat humans and animals suffering from heat-related illnesses.

In summary, HS represents a major environmental hazard compromising both human health and animal agriculture. In this study, we demonstrated that heat-stressed pigs experience increased basal insulin secretion and whole-body insulin sensitivity, and both variables likely prevent adipose tissue mobilization. The similarities with our observations during both growth and lactation in monogastrics and ruminants, coupled with the thermal therapy and diabetic models suggest that these responses are conserved amongst species, highlighting its importance in the adaptation to a thermal load. A better understanding of the physiological effects of HS is essential in order to develop treatment protocols and mitigation strategies both for human health and animal agriculture.

## References

[b1] Alamer M (2011). The role of prolactin in thermoregulation and water balance during heat stress in domestic ruminants. Asian J. Anim. Vet. Adv.

[b2] Baumgard LH, Rhoads RP (2013). Effects of heat stress on postabsorptive metabolism and energetics. Annu. Rev. Anim. Biosci.

[b3] Baumgard LH, Wheelock JB, Sanders SR, Moore CE, Green HB, Waldron MR (2011). Postabsorptive carbohydrate adaptations to heat stress and monensin supplementation in lactating Holstein cows. J. Dairy Sci.

[b4] Bouchama A, Knochel JP (2002). Heat stroke. N. Engl. J. Med.

[b5] Brelje TC, Sorenson RL (1991). Role of prolactin versus growth hormone on islet B-cell proliferation in vitro: implications for pregnancy. Endocrinology.

[b6] Brockman RP, Laarveld B (1986). Hormonal regulation of metabolism in ruminants; a review. Livest. Prod. Sci.

[b7] Burdick Sanchez NC, Chaffin R, Carroll JA, Chase CC, Coleman SW, Spiers DE (2013). Heat-tolerant versus heat-sensitive Bos taurus cattle: influence of air temperature and breed on the metabolic response to a provocative immune challenge. Domest. Anim. Endocrinol.

[b8] Changnon SA, Kunkel KE, Reinke BC (1996). Impacts and responses to the 1995 heat wave: a call to action. B. Am. Meteorol. Soc.

[b9] Chung J, Nguyen AK, Henstridge DC, Holmes AG, Chan MH, Mesa JL (2008). HSP72 protects against obesity-induced insulin resistance. Proc. Natl. Acad. Sci. USA.

[b10] Federation of Animal Sciences Societies (2010). http://www.fass.org.

[b11] Fuquay JW (1981). Heat stress as it affects animal production. J. Anim. Sci.

[b12] Furuyama T, Kitayama K, Yamashita H, Mori N (2003). Forkhead transcription factor FOXO1 (FKHR)-dependent induction of PDK4 gene expression in skeletal muscle during energy deprivation. Biochem. J.

[b13] Gaidhu MP, Bikopoulos G, Ceddia RB (2012). Chronic AICAR-induced AMP-kinase activation regulates adipocyte lipolysis in a time-dependent and fat depot-specific manner in rats. Am. J. Physiol. Cell Physiol.

[b14] Gupte AA, Bomhoff GL, Swerdlow RH, Geiger PC (2009). Heat treatment improves glucose tolerance and prevents skeletal muscle insulin resistance in rats fed a high-fat diet. Diabetes.

[b15] Hall DM, Baumgardner KR, Oberley TD, Gisolfi CV (1999). Splanchnic tissues undergo hypoxic stress during whole body hyperthermia. Am. J. Physiol.

[b16] Harris RA, Bowker-Kinley MM, Huang B, Wu P (2002). Regulation of the activity of the pyruvate dehydrogenase complex. Adv. Enzyme Regul.

[b17] Hooper PL (1999). Hot-tub therapy for type 2 diabetes mellitus. N. Engl. J. Med.

[b18] Hughes E, Huang C (2011). Participation of Akt, menin, and p21 in pregnancy-induced beta-cell proliferation. Endocrinology.

[b19] Iguchi M, Littmann AE, Chang SH, Wester LA, Knipper JS, Shields RK (2012). Heat stress and cardiovascular, hormonal, and heat shock proteins in humans. J. Athl. Train.

[b20] Kahl S, Elsasser TH, Blum JW (2000). Effect of endotoxin challenge on hepatic 5’-deiodinase activity in cattle. Domest. Anim. Endocrinol.

[b21] Kahl S, Elsasser TH, Sartin JL, Fayer R (2002). Effect of progressive cachectic parasitism and growth hormone treatment on hepatic 5’-deiodinase activity in calves. Domest. Anim. Endocrinol.

[b22] Kim JW, Dang CV (2006). Cancer's molecular sweet tooth and the Warburg effect. Cancer Res.

[b23] Kokura S, Adachi S, Manabe E, Mizushima K, Hattori T, Okuda T (2007). Whole body hyperthermia improves obesity-induced insulin resistance in diabetic mice. Int. J. Hyperthermia.

[b24] Kondo T, Sasaki K, Matsuyama R, Morino-Koga S, Adachi H, Suico MA (2012). Hyperthermia with mild electrical stimulation protects pancreatic beta-cells from cell stresses and apoptosis. Diabetes.

[b25] Lambert GP, Gisolfi CV, Berg DJ, Moseley PL, Oberley LW, Kregel KC (2002). Selected contribution: hyperthermia-induced intestinal permeability and the role of oxidative and nitrosative stress. J. Appl. Physiol.

[b26] Larson RL (2005). Effect of cattle disease on carcass traits. J. Anim. Sci.

[b27] Leon LR, Helwig BG (2010). Heat stroke: role of the systemic inflammatory response. J. Appl. Physiol. (1985).

[b28] Lu YC, Yeh WC, Ohashi PS (2008). LPS/TLR4 signal transduction pathway. Cytokine.

[b29] Masoro EJ (1977). Lipids and lipid metabolism. Annu. Rev. Physiol.

[b30] Mullur R, Liu YY, Brent GA (2014). Thyroid hormone regulation of metabolism. Physiol. Rev.

[b31] National Research Council (2012). Nutrient requirements of swine.

[b32] Niu CS, Lin MT, Liu IM, Cheng JT (2003). Role of striatal glutamate in heatstroke-induced damage in strepto-zotocin-induced diabetic rats. Neurosci. Lett.

[b33] O'Brien MD, Rhoads RP, Sanders SR, Duff GC, Baumgard LH (2010). Metabolic adaptations to heat stress in growing cattle. Domest. Anim. Endocrinol.

[b34] Pearce SC, Gabler NK, Ross JW, Escobar J, Patience JF, Rhoads RP (2013a). The effects of heat stress and plane of nutrition on metabolism in growing pigs. J. Anim. Sci.

[b35] Pearce SC, Mani V, Weber TE, Rhoads RP, Patience JF, Baumgard LH (2013b). Heat stress and reduced plane of nutrition decreases intestinal integrity and function in pigs. J. Anim. Sci.

[b36] Prather RS, Lorson M, Ross JW, Whyte JJ, Walters E (2013). Genetically engineered pig models for human diseases. Annu. Rev. Anim. Biosci.

[b37] Pucci E, Chiovato L, Pinchera A (2000). Thyroid and lipid metabolism. Int. J. Obes. Relat. Metab. Disord.

[b38] Ronchi B, Bernabucci U, Lacetera N, Verini Supplizi A, Nardone A (1999). Distinct and common effects of heat stress and restricted feeding on metabolic status in Holstein heifers. Zootec. Nutr. Anim.

[b39] Sanders SR, Cole LC, Flann KL, Baumgard LH, Rhoads RP (2009). Effects of acute heat stress on skeletal muscle gene expression associated with energy metabolism in rats. FASEB J.

[b40] Sanz Fernandez MV, Pearce SC, Gabler NK, Patience JF, Wilson ME, Socha MT (2014). Effects of supplemental zinc amino acid complex on gut integrity in heat-stressed growing pigs. Animal.

[b41] Sanz-Fernandez MV, Pearce SC, Upah NC, Long LR, Nayeri A, Sucu E (2012). Prolactin's role during acute and chronic heat stress in growing pigs. FASEB J.

[b42] Seibert J, Boddicker R, Koltes J, Reecy J, Nettleton D, Lucy M (2014).

[b43] Semenza JC, McCullough JE, Flanders WD, McGeehin MA, Lumpkin JR (1999). Excess hospital admissions during the July 1995 heat wave in Chicago. Am. J. Prev. Med.

[b44] Shwartz G, Rhoads ML, VanBaale MJ, Rhoads RP, Baumgard LH (2009). Effects of a supplemental yeast culture on heat-stressed lactating Holstein cows. J. Dairy Sci.

[b45] Stowell R, Mader T, Gaughan J (2009). Environmental Management. Livestock energetics and thermal environmental management.

[b46] St-Pierre NR, Cobanov B, Schnitkey G (2003). Economic losses from heat stress by US livestock industries. J. Dairy Sci.

[b47] Torlinska T, Banach R, Paluszak J, Gryczka-Dziadecka A (1987). Hyperthermia effect on lipolytic processes in rat blood and adipose tissue. Acta. Physiol. Pol.

[b48] Vernon RG (1992). Effects of diet on lipolysis and its regulation. P. Nutr. Soc.

[b49] Vives-Pi M, Somoza N, Fernandez-Alvarez J, Vargas F, Caro P, Alba A (2003). Evidence of expression of endotoxin receptors CD14, toll-like receptors TLR4 and TLR2 and associated molecule MD-2 and of sensitivity to endotoxin (LPS) in islet beta cells. Clin. Exp. Immunol.

[b50] Wallace TM, Levy JC, Matthews DR (2004). Use and abuse of HOMA modeling. Diabetes Care.

[b51] Wheelock JB, Rhoads RP, Vanbaale MJ, Sanders SR, Baumgard LH (2010). Effects of heat stress on energetic metabolism in lactating Holstein cows. J. Dairy Sci.

[b52] Won S, Xie G, Boddicker R, Rhoades J, Scheffler T, Scheffler J (2012). Acute duration heat stress alters expression of cellular bioenergetic-associated genes in skeletal muscle of growing pigs. J. Anim. Sci.

[b53] Zimmermann R, Strauss JG, Haemmerle G, Schoiswohl G, Birner-Gruenberger R, Riederer M (2004). Fat mobilization in adipose tissue is promoted by adipose triglyceride lipase. Science.

